# A Robust Approach for Identification of Cancer Biomarkers and Candidate Drugs

**DOI:** 10.3390/medicina55060269

**Published:** 2019-06-11

**Authors:** Md. Shahjaman, Md. Rezanur Rahman, S. M. Shahinul Islam, Md. Nurul Haque Mollah

**Affiliations:** 1 Department of Statistics, Begum Rokeya University, Rangpur 5404, Bangladesh; 2 Department of Biochemistry and Biotechnology, School of Biomedical Science, Khwaja Yunus Ali University, Sirajgonj 6751, Bangladesh; rezanur12@yahoo.com; 3 Institutitute of Biological Science (IBSC), University of Rajshahi, Rajshahi 6205, Bangladesh; shahin_ibsc@ru.ac.bd; 4 Laboratory of Bioinformatics, University of Rajshahi, Rajshahi 6205, Bangladesh

**Keywords:** candidate drugs, cancer biomarkers, DEGs, FC, *p*-value, paired samples, minimum *β*-divergence estimation, robustness

## Abstract

*Background and objectives:* Identification of cancer biomarkers that are differentially expressed (DE) between two biological conditions is an important task in many microarray studies. There exist several methods in the literature in this regards and most of these methods designed especially for unpaired samples, those are not suitable for paired samples. Furthermore, the traditional methods use *p*-values or fold change (FC) values to detect the DE genes. However, sometimes, *p*-value based results do not comply with FC based results due to the smaller pooled variance of gene expressions, which occurs when variance of each individual condition becomes smaller. There are some methods that combine both *p*-values and FC values to solve this problem. But, those methods also show weak performance for small sample cases in the presence of outlying expressions. To overcome this problem, in this paper, an attempt is made to propose a hybrid robust SAM-FC approach by combining rank of FC values and rank of *p*-values computed by SAM statistic using minimum *β*-divergence method, which is designed for paired samples. *Materials and Methods*: The proposed method introduces a weight function known as *β*-weight function. This weight function produces larger weights corresponding to usual and smaller weights for unusual expressions. The *β*-weight function plays the significant role on the performance of the proposed method. The proposed method uses *β*-weight function as a measure of outlier detection by setting *β* = 0.2. We unify both classical and robust estimates using *β*-weight function, such that maximum likelihood estimators (MLEs) are used in absence of outliers and minimum *β*-divergence estimators are used in presence of outliers to obtain reasonable *p*-values and FC values in the proposed method. *Results:* We examined the performance of proposed method in a comparison of some popular methods (*t*-test, SAM, LIMMA, Wilcoxon, WAD, RP, and FCROS) using both simulated and real gene expression profiles for both small and large sample cases. From the simulation and a real spike in data analysis results, we observed that the proposed method outperforms other methods for small sample cases in the presence of outliers and it keeps almost equal performance with other robust methods (Wilcoxon, RP, and FCROS) otherwise. From the head and neck cancer (HNC) gene expression dataset, the proposed method identified two additional genes (CYP3A4 and NOVA1) that are significantly enriched in linoleic acid metabolism, drug metabolism, steroid hormone biosynthesis and metabolic pathways. The survival analysis through Kaplan–Meier curve revealed that combined effect of these two genes has prognostic capability and they might be promising biomarker of HNC. Moreover, we retrieved the 12 candidate drugs based on gene interaction from glad4u and drug bank literature based gene associations. *Conclusions:* Using pathway analysis, disease association study, protein–protein interactions and survival analysis we found that our proposed two additional genes might be involved in the critical pathways of cancer. Furthermore, the identified drugs showed statistical significance which indicates that proteins associated with these genes might be therapeutic target in cancer.

## 1. Introduction

One of the important and common objectives of microarray experiments is to detect the genes that can differentiate biological samples into two biological conditions. These genes are called differentially expressed genes (DEGs). Biomarkers are the few DEGs that have prognostic capability of a specific disease. In the earlier days of microarray experiments, the simplest method fold change (FC) was used to identify the DEGs or EEGs (equally expressed genes) [1,2]. In this method, log-ratio of gene expressions between two conditions is calculated and a particular gene is said to be DEG, for which the absolute value of FC exceeds an arbitrary cut-off value that depends on user interest. There are several FC based methods for DEGs selection such as average difference (AD), weighted average difference (WAD), FC, and distributional fold change (DFC) [3,4].

On the other hand, there are two types of statistical approaches based on *p*-values to select the DEGs. One is parametric approach and the other is nonparametric approach. In parametric approach, the *t*-test and *t*-test-like approaches are typically used to identify the DEGs. However, the conventional *t*-test suffers from two types of drawbacks: One is multiple testing problems, for example, in microarray data having thousands of genes the *t*-test needs to be performed thousands of times, separately. Therefore, some genes with large t-statistic are false selected as DEGs because of the low variance of gene expressions. Another drawback of the *t*-test occurs with small sample sizes, which implies low statistical power. The extended version of *t*-test-like significance analysis of microarrays (SAM) and linear models for microarray (LIMMA) overcomes the shortcomings of *t*-test [5,6,7,8]. The analysis of variance (ANOVA) is widely used to microarray data to identify genes with more than two conditions [9,10]. The parametric approaches often rely on the assumption of normality of the data. While nonparametric approaches like Kruskal–Wallis (KW), Wilcoxon test [11,12], and SAM do not rely on such assumptions. Nowadays, researchers are also interested to finding cluster-based DEGs [13].

However, most of the methods discussed earlier are suitable only for the unpaired independent samples to detect DEGs. They are not suitable for the datasets generated from the paired correlated samples to detect DEGs [14,15]. Due to the heterogeneity of cancer genetics, paired samples measurements were used to detect DEGs by few researchers [16,17]. In paired data analysis, the repeated measurements within the subjects are considered rather than the across subjects.

Moreover, most of the statistical methods discussed earlier control type I (false-positive) error rate such as family-wise error rate (FWER) or false discovery rate (FDR) to avoid multiple testing problem [18]. However, they do not give much attention on controlling type II (false-negative) error rate which usually occur in presence of outliers with low expressions of DEGs. Thus the researchers may lose some important DEGs by the existing methods since they are not robust against outliers. Parametric approaches, like hybrid ANOVA [19], and nonparametric approaches, like KW [11], are robust against outliers for large sample cases, but they show weak performance in small sample cases.

Furthermore, all the methods discussed earlier make the decision of DEGs independently by their own criteria. The *p*-value-based statistical tests sometimes detect significant DEGs with small fold changes due to smaller pooled variance of the gene expressions, which occurs when variance for each individual condition becomes smaller. However these small fold change values may not be biologically meaningful [20]. So a contradiction between statistical and biological point of view is created to select the DEGs. To overcome this problem, some methods were proposed that take variability of the gene expressions level into account with FC information such as rank products (RP), *t*-tests relative to a threshold (TREAT), *π*-value, and fold change rank ordering statistics (FCROS) [20,21,22,23]. However these methods show weak performance in small samples in the presence of outliers. Therefore, in this paper an attempt is made to develop a hybrid robust SAM-FC approach by combining rank of FC values and rank of *p*-values based on SAM statistic using minimum *β*-divergence method to overcome all the aforementioned problems. The proposed method is designed for paired samples and can also be applied for equal sample size of two conditions. The formulation of the proposed algorithm will be discussed in the next section and the performance of the proposed algorithm in a comparison of the other methods as early mentioned using both simulated and real gene expression profiles of cancer diseases will be discussed in the results and discussion section.

## 2. Materials and Methods

Let *x_gik_* be the *g*th gene expression values of *k*th replicates in the *i*th condition (*g* = 1, 2, ..., *G*; *i* = 1,2; *k* = 1, 2, ..., *n*; *n* = *n*_1_ = *n*_2_), which follows normal distribution with mean *µ_gi_* and variance *σ*^2^. That is

(1) $$x_{gik}\sim N\left(\mu_{gi},\sigma^{2}\right)$$

Then the FC values of the *g*th gene is denoted as *d*_gk_ = *x*_g1k_ − *x*_g2k_ (*k* = 1, 2, ..., *n*) and also follow *N* (*µ_g_*, *σ_g_*^2^); where, *µ_g_* = *µ_g_*_1_ − *µ_g_*_2_ and *σ_g_*^2^ > 0, respectively. Now we want to test

(2) $$H_{0}:\mu_{g}=0\;\mathrm{vs}\;H_{1}:\mu_{g}\neq 0$$

Which implies that $H_{0}:\mu_{g1}=\mu_{g2}\;\mathrm{vs}\;H_{1}:\mu_{g1}\neq \mu_{g2}$ assuming that *σ_g_*^2^ > 0 is unknown. If *H*_0_ is accepted, the *g*th gene is said to be equally expressed (EE) gene; otherwise it is DE gene. The simple statistical method for detecting DE gene from the paired samples is the *t*-test. The formula for the one sample *t*-test is as follows

(3) $$t_{g}=\frac{r_{g}}{s_{g}/\sqrt{n}};g=1,2,\ldots ,G$$

Which follows the Student’s *t* distribution with (*n* − 1) degrees of freedom. Where, $r_{g}$ and $s_{g}$ are the estimates of *µ_g_* and *σ_g_*, respectively. However the identification of DEGs using a *t*-test is highly sensitive to outliers, and it also shows poor performance when the sample size is small. The SAM based on modified *t*-statistic overcomes the shortcomings of the *t*-test in microarray experiments with small sample sizes [7]. The SAM algorithm for paired samples was developed with the following modifications of the *t*-statistic.

(4) $$r_{g}={\hat{\mu }}_{g}=\frac{\sum_{k=1}^{n}d_{gk}}{n}$$

(5) $$s_{g}={\hat{\sigma }}_{g}={\left\{\frac{\sum_{k=1}^{n}{\left(d_{gk}-{\hat{\mu }}_{g}\right)}^{2}}{\left(n-1\right)}\right\}}^{1/2}$$

(6) $$u_{g}=\frac{r_{g}}{s_{g}^{*}+s_{0}};g=1,2,\ldots ,G$$

where, $s_{g}^{*}=\frac{s_{g}}{\sqrt{n}}$ and *s*_0_ represent the percentile of the distribution of sample standard deviations, which is chosen in a way so that the coefficient of variation of $u_{g}$ is minimized. However, this modified statistic (6) for SAM is also sensitive to outliers. Therefore, in this paper, we update Equation (6) using minimum *β*-divergence estimators instead of maximum likelihood estimators (MLEs) in the presence of outliers. The minimum *β*-divergence estimators ${\hat{\theta }}_{g,\beta }=\left({\hat{\mu }}_{g,\beta },{\hat{\sigma }}_{g,\beta }^{2}\right)$ of the parameter $\theta_{g}=\left(\mu_{g},\sigma_{g}^{2}\right)$ are obtained iteratively as follows

(7) $$\mu_{g,t+1}=\frac{\sum_{k=1}^{n}\emptyset_{\beta }(d_{gk}|\theta_{g,t})d_{gk}}{\sum_{k=1}^{n}\emptyset_{\beta }(d_{gk}|\theta_{g,t})}$$

(8) $$\sigma_{g,t+1}^{2}=\frac{\sum_{k=1}^{n}\emptyset_{\beta }\left(d_{gk}|\theta_{g,t}\right){\left(d_{gk}-\mu_{g,t}\right)}^{2}}{{\left(\beta +1\right)}^{-1}\sum_{k=1}^{n}\emptyset_{\beta }(d_{gk}|\theta_{g,t})}$$

where,

(9) $$\emptyset_{\beta }\left(d_{gk}|\theta_{g}\right)=exp\left\{-\frac{\beta }{2\sigma_{g}^{2}}{\left(d_{gk}-\mu_{g}\right)}^{2}\right\}$$

which is known as *β*-weight function [24,25]. This *β*-weight function (9) produces larger weights (≤1) corresponding to usual/normal expressions and smaller weights (≥0) corresponding to unusual/outlying expressions of *d_gk_*. Note that the minimum *β*-divergence estimators ${\hat{\theta }}_{g,\beta }=\left({\hat{\mu }}_{g,\beta },{\hat{\sigma }}_{g,\beta }^{2}\right)$ reduce to MLEs ${\hat{\theta }}_{g}=\left({\hat{\mu }}_{g},{\hat{\sigma }}_{g}^{2}\right)$ for *β* = 0. Since the MLEs of Gaussian distribution are consistent and asymptotically efficient, we used MLEs in Equation (6) in the absence of outliers. We employ the *β*-weight function (9) as a measure of outlier detection by setting *β* = 0.2. The robustness and efficiency of the estimators depends on the tuning parameter *β*. Therefore, it is very important to select *β* properly. There are several existing approaches for selection of tuning parameter *β* including leave-one-out (LOOCV) and *K*-fold cross-validation (CV) [24,25]. In the absence and presence of outliers, the CV approach produces *β* = 0 and *β* > 0, respectively. The minimum *β*-divergence estimators in Equations (7) and (8) reduce to MLEs in Equations (4) and (5) with *β* = 0, as earlier mentioned. So we can apply the CV approach. However, this approach needs to execute thousands of time since microarray data usually contains thousands of genes. Therefore, CV approach may prove conservative in this case. For this reason, in this paper we consider outlier detection approach based on *β*-weight function with *β* = 0.2. This kind of *β* selection was also used in Shahjaman et al. (2017) [26].

Fold change gene expression, *d_gk_*, is categorized by the *β*-weight function with *β* = 0.2 as follows

(10) $$\emptyset_{\beta }\left(d_{gk}|{\hat{\theta }}_{g,\beta }\right)=\{\begin{array}{c} >\delta ,\;\mathrm{if}\;d_{gk}\;\mathrm{is}\;\mathrm{not}\;\mathrm{an}\;\mathrm{outlier}\\ \leq \delta ,\;\mathrm{if}\;d_{gk}\;\mathrm{is}\;\mathrm{an}\;\mathrm{outlier} \end{array}$$

where, the $\delta_{0}$ is determined by the following equation.

(11) $$\delta =\min(0{.2,\delta }_{0})$$

(12) $$\delta_{0}=\min\left(\emptyset_{\beta }\left(d_{gk}|{\hat{\theta }}_{g,\beta }\right)\right)+\alpha \left[\max\left(\emptyset_{\beta }\left(d_{gk}|{\hat{\theta }}_{g,\beta }\right)\right)-\min\left(\emptyset_{\beta }\left(d_{gk}|{\hat{\theta }}_{g,\beta }\right)\right)\right]\forall g,k$$

Here, α=0.1. Thus we unify the MLEs and the minimum *β*-divergence estimators as follows

(13) $${\hat{\theta }}_{g,\beta }=\{\begin{array}{c} {\hat{\theta }}_{g,\beta },\;\mathrm{if}\;\sum_{k=1}^{n}I_{\left[\emptyset_{\beta }\left(d_{gk}|{\hat{\theta }}_{g,\beta }\right)>\delta \right]}<n\\ {\hat{\theta }}_{g},\;\mathrm{if}\;\sum_{k=1}^{n}I_{\left[\emptyset_{\beta }\left(d_{gk}|{\hat{\theta }}_{g,\beta }\right)>\delta \right]}=n \end{array}$$

Here $\sum_{k=1}^{n}I_{\left[\emptyset_{\beta }\left(d_{gk}|{\hat{\theta }}_{g,\beta }\right)>\delta \right]}=n$ indicates that *g*th gene expressions are not contaminated by outliers and $\sum_{k=1}^{n}I_{\left[\emptyset_{\beta }\left(d_{gk}|{\hat{\theta }}_{g,\beta }\right)>\delta \right]}<n$ indicates that at least one expression of *g*th gene is contaminated by outliers. We replace $r_{g}$ and $s_{g}$ in Equation (6) with ${\hat{\mu }}_{g,\beta }$ and ${\hat{\sigma }}_{g,\beta }^{}$, respectively, which allows for calculation of the reasonable *p*-values for testing the null hypothesis (2) using the following test statistic, which we call *β*-SAM statistic. Note that this *β*-SAM statistic ($u_{g,\beta }$) reduces to the classical SAM statistic ($u_{g}$) for *β* = 0.

(14) $$u_{g,\beta }=\frac{{\hat{\mu }}_{g,\beta }}{\frac{{\hat{\sigma }}_{g,\beta }^{}}{\sqrt{n}}+s_{0}};g=1,2,\ldots ,G$$

Now we calculate the *β*-FC values for the *g*th gene as follows

(15) $${\mathrm{FC}}_{g,\beta }=\{\begin{array}{c} {\hat{\mu }}_{g,\beta },\;\mathrm{in}\;\mathrm{presence}\;\mathrm{of}\;\mathrm{outliers}\\ {\hat{\mu }}_{g},\;\mathrm{in}\;\mathrm{absence}\;\mathrm{of}\;\mathrm{outliers}\; \end{array}$$

Which reduces to the classical FC as ${\mathrm{FC}}_{g}={\hat{\mu }}_{g}$ for *β* = 0.

### 2.1. Selection of Top Differential Gene Expressions

Our proposal is to select the top DEGs by combining both the rank of *β*-FC and rank of *β*-SAM values using the following steps.

1. Calculate *β*-FC and *β*-SAM values for each gene.
2. Rank both |*β*-FC| and |*β*-SAM| values separately in descending order, where |*β*-FC| indicates the absolute value of *β*-FC.
3. Calculate the average of both ranks for each gene.
4. Arrange the average rank values in ascending order.
5. Select first *N* ordered genes as top DE genes such that *β*-SAM of *N*th ordered gene produces *p*-value < 0.1.

### 2.2. Performance Assessment

Let us consider a two-class prediction problem such as DEG or EEG. The outcomes are then divided into four categories: (i) DEGs are correctly predicted as DEGs (true positives: TP), (ii) DEGs are incorrectly predicted as EEGs (false-negatives: FN), (iii) EEGs are correctly predicted as EEGs (true negatives: TN), and (iv) EEGs are incorrectly predicted as DEGs (false-positives: FP). To investigate the performance of algorithms for detection of differential gene expression, we employed the following measures using the above categories.

(16) $$\mathrm{True}\;\mathrm{Positive}\;\mathrm{Rate}\;(\mathrm{TPR})\;=\frac{n\left(TP\right)}{\;n\left(TP\right)+n\left(FP\right)}$$

(17) $$\mathrm{True}\;\mathrm{Negative}\;\mathrm{Rate}\;(\mathrm{TNR})=\frac{n\left(TN\right)}{n\left(TN\right)+n\left(FP\right)}$$

(18) $$\mathrm{False}-\mathrm{Positive}\;\mathrm{Rate}\;(\mathrm{FPR})=\frac{n\left(FP\right)}{n\left(TN\right)+n\left(FP\right)}$$

(19) $$\mathrm{False}-\mathrm{Negative}\;\mathrm{Rate}\;(\mathrm{FNR})=\frac{n\left(FN\right)}{n\left(TP\right)+n\left(FN\right)}$$

(20) $$\mathrm{False}\;\mathrm{Discovery}\;\mathrm{Rate}\;(\mathrm{FDR})=\frac{n\left(FP\right)}{n\left(TP\right)+n\left(FP\right)}$$

(21) $$\mathrm{False}\;\mathrm{Omission}\;\mathrm{Rate}\;(\mathrm{FOR})=\frac{n\left(TN\right)}{n\left(TN\right)+n\left(FN\right)}$$

(22) $$\mathrm{Misclassification}\;\mathrm{Error}\;\mathrm{Rate}\;(\mathrm{MER})=\frac{n\left(FP\right)+n\left(FN\right)}{n\left(TP\right)+n\left(FP\right)+n\left(FP\right)+n\left(FN\right)}$$

where, *n*(*TN*) denotes the number of true negatives and so on. We used receiver operating characteristic (ROC) curve (FPR vs. TPR), area under the ROC curve (AUC), and partial AUC (pAUC) to evaluate the performance of the methods. Each of these performance measures produces values between 0 to 1. A method is said to be best performer if it produces largest values of TPR, TNR, AUC, and pAUC and the smallest values of FPR, FNR, FOR, FDR, and MER.

## 3. Results

We demonstrated the performance of the proposed method in a comparison with other popular existing methods, such as *t*-test [9], SAM [7], Wilcoxon [12], and LIMMA [5], and fold change-based methods WAD [4], RP [21], and FCROS [22], using both simulated and real gene expression data. We used six R packages of others methods such as stats, *samr* [8], TCC [27], *fcros* [22], *limma* [6], and *RankProd* [21]. We employed all the seven methods including our proposed method in the one simulated and two microarray gene expression datasets to evaluate the performance of these methods. The description of these datasets is as follows.

Data Type 1: Simulated Gene Expression Profiles

We generated 100 datasets from the model described in equation (1) for both small (*n*_1_ = *n*_2_ = 3) and large sample (*n*_1_ = *n*_2_ = 15) cases, respectively, for the arbitrary values of (*µ*_1_, *µ*_2_) ∈ (3, 5) and *σ*^2^ = 0.05. Each dataset represented the gene expression profiles for 10,000 genes with (*n* = *n*_1_ = *n*_2_) samples. Among the 10,000 genes represented in each dataset, the numbers of DEGs were set to 300 (proportion of DEGs = 0.03) and the other 9700 genes were considered EEGs. To investigate the robustness performance of all the methods, we contaminated each of 100 dataset by outliers. In the contaminated dataset, we contaminated one or two expressions for each gene across the genome out of (*n*_1_ + *n*_2_) expressions by outliers. We generated an outlying expression in the *i*th condition for *g*th gene using $x_{gik}^{*}=d+2\times \max(x_{gik};k=1,2,\ldots ,n_{i};i=1,2;$ where, d ∈ (5, 10) is an arbitrary value. Then we replace $x_{gik}$ by $x_{gik}^{*}$ in the data matrix to get the contaminated dataset.

Data Type 2: Platinum Spike Gene Expression Profiles

This dataset consists of 18 spike-in samples with 9 controls versus 9 tests [28]. This dataset can be downloaded from the Gene Expression Omnibus website under the accession number GSE21344. Using RMA preprocessing and filtering of this dataset, we retained 18,707 probes, among which 1944 probes are known as the DEGs under spiked-in fold changes of 0.25, 0.28, 0.40, 0.66, 0.83, 1.5, 1.7, 2, 3, and 3.5. To investigate the performance of the proposed method in a comparison of the other methods in presence of outliers with the real dataset, we contaminated this dataset by outliers, where outliers are generated as data type 1.

Data Type 3: Head and Neck Cancer Gene Expression Profiles

Gene expression profiles were collected from 42 paired (from the same patient) samples of head and neck squamous cell carcinoma (HNSCC) and normal tissue. We used the normalized head and neck cancer (HNC) dataset [29]. We downloaded this dataset from GEO website with accession number GSE663. After preprocessing of RNA samples using Affymetrix GeneChip CELL files were obtained. Further, Robust Multichip Analysis (RMA) was used to obtain signal to probes from the CELL file. This dataset contains 12,642 probe sets.

### 3.1. Performance Evaluation Based on Simulated Gene Expression Profiles

**Figure 1:**
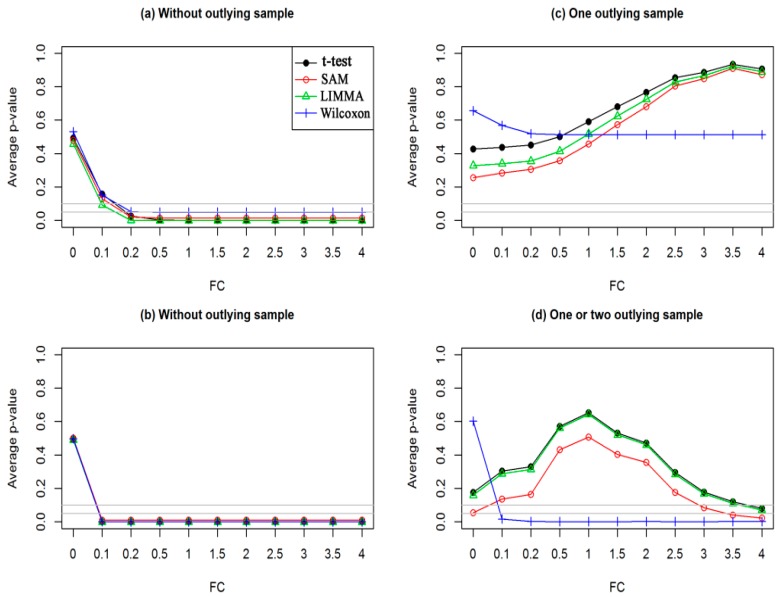
Plot of average *p*-values against FC values by different test procedures for simulated dataset. For small sample cases (*n*_1_ = *n*_2_ = 3) (**a**) without outlying sample and (**c**) one outlying sample. For large sample cases (*n*_1_ = *n*_2_ = 15): (**b**) without outlying sample and (**d**) one or two outlying samples.

**Figure 2:**
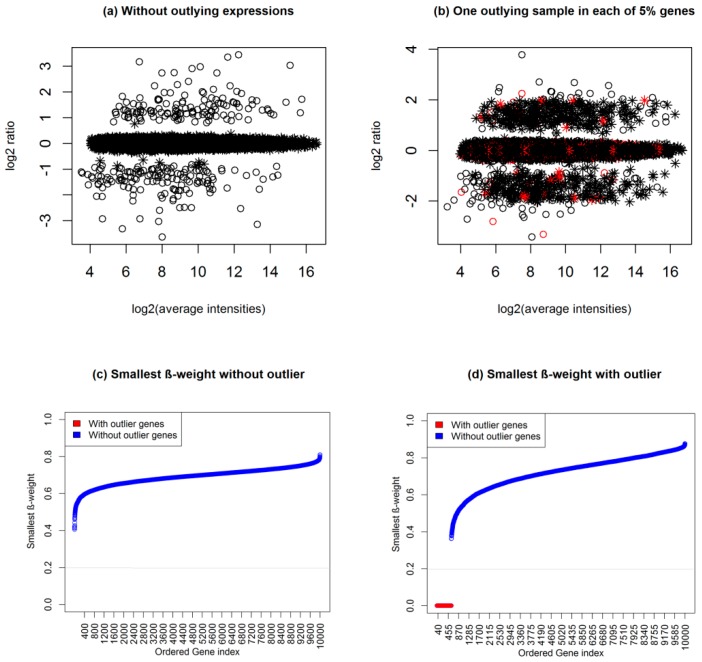
M-A Plot and scatter plot of *β*-weights using data type 1 for small sample case (*n*_1_ = *n*_2_ = 3). (**a**) Without outlying sample. (**b**) One outlying sample in each of 5% genes. (**c**) Scatter plot of the smallest *β*-weight for (a). (**d**) Scatter plot of the smallest *β*-weight for (b); where we considered the minimum value of *β*-weights of *n* = 3 fold change expressions corresponding to *n*_1_ = 3 samples of first condition and *n*_2_ = 3 samples of second condition as the smallest *β*-weight.

Sometimes, statistical methods select DEGs who have no biological significance (genes with small fold changes). Hence, researchers now require genes who satisfy both statistical significance (*p*-value < 0.05) and biological significance (absolute FC > 1.5 or 2). For example Mollah et al. (2015) declared genes to be DEGs if they show absolute FC > 2 and also satisfy adjusted *p*-value < 0.05 [19]. Xiao et al. (2014) introduced a score that combine both the FC and *p*-value together [23]. They found that this combination of gene ranking provided much better results than only *p*-value-based ranking. However, sometimes, existing methods fail to meet their interests due to smaller variance of gene expressions or in presence of outliers. For example, a gene satisfying 0 < absolute FC < 1.5 may declared as DEG with adjusted *p*-value < 0.05 obtained by the existing popular statistical methods (*t*-test, SAM, LIMMA, and Wilcoxon) in absence of outliers for both small-and large sample cases (see Figure 1a,b), which leads to increase the FDR. On the other hand, a gene with absolute FC > 1.5 may be declared as EEG with adjusted *p*-value > 0.2 in presence of outliers for both small-and large sample cases (see Figure 1c,d), which leads to increase the false omission rate (FOR) by each of the four methods, except KW for large sample cases. The other methods that combine *p*-value and FC value are [20,21,22,23], but they show weak performance when the sample size is low in the presence of outliers. We can also visualize the DEGs by considering both *p*-value and FC value using Volcano plot [30]. In this plot minus log10(*p*-value) is plotted against log2FC and usually the genes showing values above certain thresholds of the two quantities are called DEGs. To demonstrate the performance of traditional SAM and the proposed robust SAM using the Volcano plot we generated a dataset using (1). This dataset consists of 1000 genes including 50 upregulated DEGs and 50 downregulated DEGs. Figure S1a in supplenetary file 1 shows the Volcano plot in presence of 10% outliers for small sample cases. In this plot the blue, green and black symbols represent the upregulated DEGs, downregulated DEGs, and EEGs, respectively. The *p*-values were calculated by the traditional SAM method. We clearly observe that there are many outlying DEGs those are not satisfy the criteria of *p*-value < 0.05 (horizontal gray dot line) and absolute FC > 1.5 (magenta vertical line). We also observe that there are few EEGs that are declared as DEGs by this criteria. Thus using the traditional SAM method we fail to detect the true DEGs in presence of outliers. Figure S1b represents the Volcano plot by our proposed method. From this plot it is evident that our proposed method can detect the true DEGs in presence of outliers by combining FC values and *p*-values. Therefore, in this paper, we developed a hybrid robust SAM-FC approach by combining rank of FC values and rank of *p*-values based on SAM statistic using the minimum *β*-divergence estimation to overcome the problems as mentioned above. Figure 2a,b represents the M-A plot of a dataset, which is one of the 100 simulated datasets of data type 1 for the small sample case (*n*_1_ = *n*_2_ = 3) in absence and presence of one outlying sample with each of 5% genes, respectively. The circle and asterisk symbols in the Figure 2a indicates that DEGs and EEGs, respectively. In Figure 2b, the red circle and red asterisk symbols indicate the outliers with DEGs and EEGs, respectively. To investigate the outlier detection performance of the proposed method, we present Figure 2c,d, corresponding to absence and presence of the outlying dataset (see Figure 2a,b), respectively. In these figures, we calculated the *β*-weights for each gene based on the n = 3 FC observations corresponding to n_1_ = 3 samples of first condition and *n*_2_ = 3 samples of second condition using the Equations (7) and (8) in Equation (9). Then we calculate the smallest *β*-weight for each gene. The predicted and observed distributions of the *β*-weights are shown in Figure S2 in the supplementary file 1. The Figure 2c,d represent the scatter plot of the smallest *β*-weight for each of 10,000 genes against their ordered gene index. The gray line (see Figure 2c,d) indicates the threshold line of the cut-off value *δ*, which is computed using (11). From Figure 2c we observed that all the smallest *β*-weights for each gene are above the threshold line with *δ* = 0.2, which indicates that there is no outlying genes in the dataset. In the Figure 2d, we see that some smallest *β*-weights (red colour) are below the threshold line with *δ* = 0.2, which indicates that the genes associated with these smallest *β*-weights are outlying genes. Thus our proposed method can detect the outliers and unify both classical and robust estimates in Equation (13).

#### Performance Evaluation Based on Simulated Gene Expression Profiles Using Data Type 1

**Figure 3:**
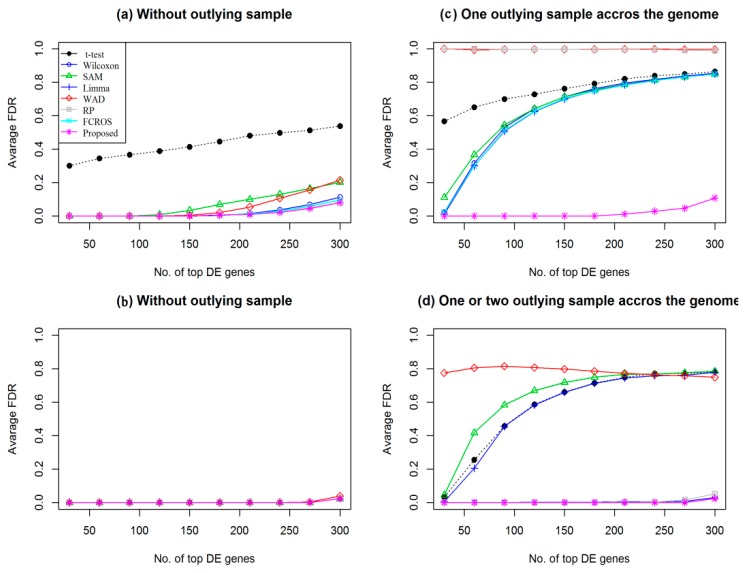
Plot of average false discovery rate (FDR) versus top 300 differentially expressed genes (DEGs) estimated by different methods using data type 1. For small sample cases (*n*_1_ = *n*_2_ = 3): (**a**) without outlying sample and (**c**) one outlying sample across the genome. For large sample cases (*n*_1_ = *n*_2_ = 15): (**b**) without outlying sample and (**d**) one or two outlying samples across the genome.

To investigate the performance of the proposed method for the detection of DEGs in a comparison with seven popular methods (*t*-test, SAM, LIMMA, Wilcoxon, WAD, RP, and FCROS), we computed average FDR corresponding to the top 300 estimated DEGs based on 100 datasets of data type 1 for each method, in absence and presence of one or two outlying expressions for each gene across the genome for both small (*n*_1_ = *n*_2_ = 3) and large sample (*n*_1_ = *n*_2_ = 15) cases, respectively (which is shown in Figure 3). The *p*-values were obtained from each of the methods are adjusted by the Benjamin–Hochberg (BH) method for multiple testing correction. We declare a gene as DEG if the adjusted *p*-value corresponding to this gene is less than 0.05. From Figure 3a,b, we observe that all the eight methods performed almost equally in the absence of outlying sample for both small and large sample cases, respectively. However, for the small sample case, our proposed method produces slightly better performance than the other methods. However in presence of outliers the proposed method produces much smaller FDR than the other methods, for small sample case (see Figure 3c). In the case of large sample (see Figure 3d) in presence of outliers, Wilcoxon, FCROS, RP, and the proposed method show better performance compare to the other methods (*t*-test, SAM, LIMMA, and WAD). We also computed the average values of different performance measures (TPR, FPR, TNR, FNR, FDR, MER, AUC, and pAUC) to construct the Table 1. In this table the results inside the parenthesis (.) indicate the summary statistics in presence of outliers. From this table we also observe that the proposed method produces much better results than the other methods since it produces larger values of TPR, TNR, AUC, and pAUC and smaller values of FNR, FPR, FDR, and MER for all the cases. Furthermore, we performed a proportion test to determine the statistical significance of several proportions produced by the eight methods for each of the performance measures. The *p*-values of Table 1 were obtained by the Pearson’s chi-squared test statistic [31]. From this results we can conclude that since all the *p*-values corresponding to each performance measures except FPR (in presence of outliers) are less than 0.01 in small samples for both in absence and presence of outliers, therefore they are highly statistically significant. In this case we also found that the FPR (*p*-value < 0.05) is statistically significant at 5% level of significance in presence of outliers. On the other hand, for large sample case in absence of outliers all the performance measures show statistically insignificant, whereas, in the presence of outliers, all the performance measures are statistically significant at the 1% level of significance. Figures S3 and S4 display the box plot of AUC and MER values based on 100 datasets of data type 1. The plot of FNR against FPR and ROC curve are also shown in Figures S5 and S6, respectively. Similar results were found from these figures like FDR plot. Thus on an average the proposed method outperforms other methods.

### 3.2. Performance Evaluation Based on Real Gene Expression Profiles

We used two microarray gene expression datasets to evaluate the performance of the proposed method in a comparison of the other methods as discussed earlier. The first dataset is the platinum spike gene expression profiles [28] (data type 2) and second dataset is the head and neck cancer gene expression profiles [29] (data type 3).

#### 3.2.1. Performance Evaluation Based on Platinum Spike Gene Expression Profiles

This dataset of data type 2 contains 18,707 probes with 18 spike samples, nine in each condition. There were 1944 genes recommended as valid DEGs under spiked-in fold changes of 0.25, 0.28, 0.40, 0.66, 0.83, 1.5, 1.7, 2, 3, and 3.5, and the other 16,743 genes were recommended as EEGs. To investigate the performance of the proposed method in a comparison of the other seven methods (*t*-test, Wilcoxon, SAM, LIMMA, WAD, RP, and FCROS) with the original and contaminated datasets, we employed each of these methods in both datasets to identify the DEGs. We computed different performance indices (TPR, TNR, FPR, FNR, FDR, MER, AUC, and pAUC) based on top 1944 estimated DEGs by each method. The DEGs were selected using adjusted *p*-values < 0.05 criterion for each of the methods. The *p*-values were adjusted by the Benjamin–Hochberg (BH) method. The summary statistics of these performance indices is presented in Table 2. The results inside the parenthesis (.) indicate the summary statistics for the contaminated dataset. We observed that all the methods produce almost similar results with the original dataset. However, the proposed method produces slightly better performance than the other methods. For example, the proposed method produces AUC (pAUC) = 0.837 (0.170), which is larger than 0.816 (0.161), 0.805 (0.158), 0.832 (0.165), 0.828 (0.164), 0.830 (0.165), 0.822 (0.163), and 0.833 (0.163), that are produced by *t*-test, Wilcoxon, SAM, LIMMA, WAD, RP, and FCROS, respectively. In addition, the proposed method produces much better results than the other seven methods for the contaminated dataset since the proposed method produces larger values of TPR, TNR, AUC, and pAUC and smaller values of FNR, FPR, FDR, and MER. To determine whether the performance indices produced by the eight methods are statistically significant or not, we conducted a proportion test for each of the performance indices. The *p*-values of Table 2 were obtained from this test statistic. From this table we observed that in absence of outliers the *p*-values for each of the performance measures show statistically insignificance (*p*-value > 0.05). This indicates that the performances of all methods are similar in absence of outliers. Whereas, in presence of outliers, they show statistically significant at 1% level of significance. This indicates that the proposed method outperformed other methods in presence of outliers. The outlier detection performance using *β*-weight function of the proposed method is demonstrated with the contaminated dataset. These results are displayed in Figure S7 in supplementary file 1. Figure S7a shows the scatter plot of the smallest *β*-weight for each gene with its index and Figure S7b show the scatter plot of ordered smallest *β*-weights corresponding to their gene index. We considered the minimum value of *β*-weights of *n* = 9 expressions as the smallest *β*-weight for each gene in both figures. Outlying genes are indicated by the red colour. Thus we observed in Figure S7a that the *β*-weight function produces smaller weights for outlying genes, where an outlying gene is defined if its smallest *β*-weight is smaller than the cut-off value, *δ* = 0.2 (see Equation (11)).

To investigate the performance of the proposed method in a comparison of the other methods for the small sample case in both original and contaminated datasets, we generated 100 bootstrap datasets from each of the dataset as discussed earlier. In each bootstrap dataset, we selected three expression data points randomly from each condition for each gene. Then we estimated the performance indices by each of the methods as before. Note that the bootstrap samples were considered to increase the accuracy and precision of the estimates. It is noticeable from Table 3 that all the methods produce similar results with the original dataset except Wilcoxon, whereas the proposed method produces much better results with the contaminated dataset. In addition, we conducted a statistical test (as before) to determine the significance of several proportions produced by the eight methods for each of the performance indices. Since all the *p*-values in Table 3 are less than 0.01, so we can conclude that the performance results are highly statistically significant. This indicates that proposed method outperformed other methods. Table S1 in the supplementary file 2 represents the comparative results of the eight methods with the valid differentially expressed (DE) gene-set in both original and contaminated datasets for sample sizes *n*_1_ = *n*_2_ = 9 and 3, respectively. These results also supported the results of Table 2; Table 3. Thus from this study we may conclude that the performance of the proposed method improves over the other methods.

#### 3.2.2. Performance Evaluation Based on Head and Neck Cancer Gene Expression Profiles

**Figure 4:**
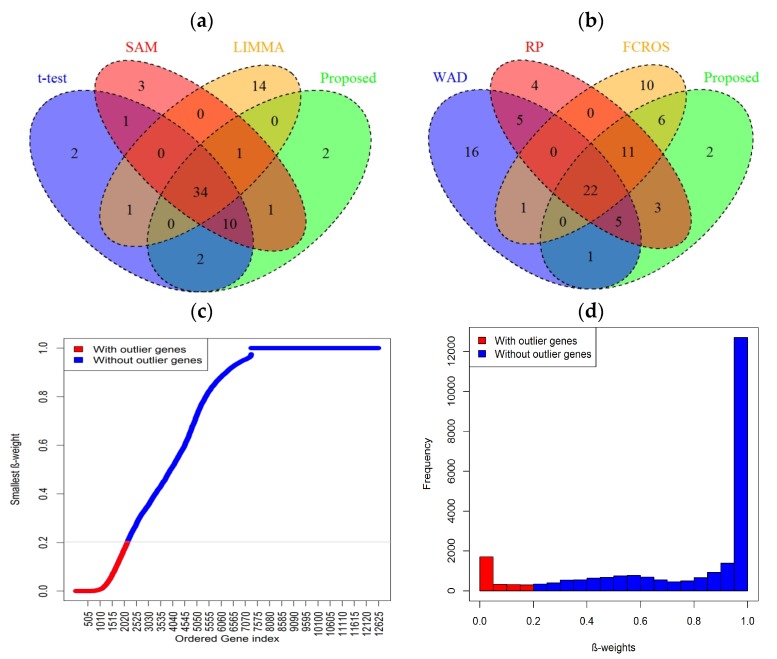
Comparison of the top selected genes by different methods for the head and neck cancer dataset. Venn diagram of top 50 genes estimated by (**a**) *t*-test, SAM, and LIMMA, and proposed or by (**b**) WAD, RP, FCROS, and proposed method. (**c**) Plot of ordered smallest *β*-weight for each gene and (**d**) histogram of *β*-weights. Where the smallest *β*-weight represents the minimum value of 22 *β*-weights for 22 paired samples for each gene. The outlier genes are indicated in red color. The gray line indicates the maximum value of cutoff, *δ* = 0.2 for outlying genes.

**Figure 5:**
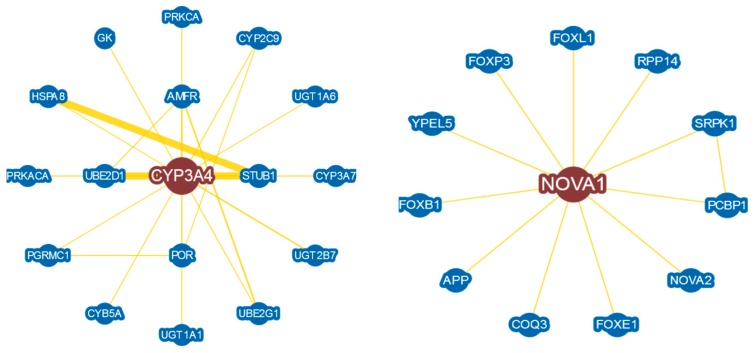
Protein–protein interaction (PPI) network using 2 genes detected by the proposed method.

**Figure 6:**
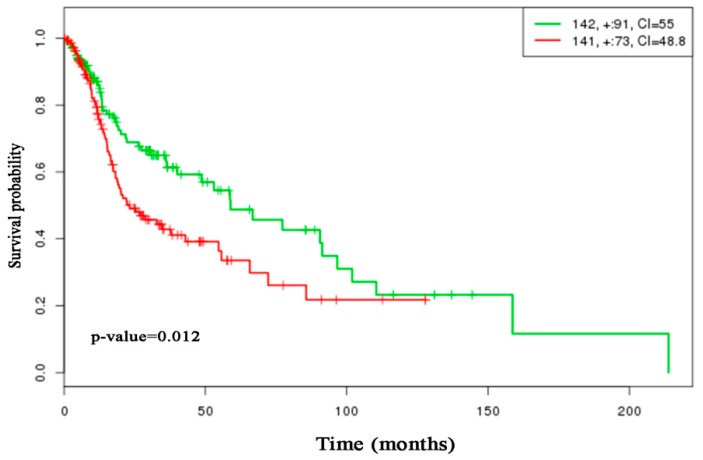
Kaplan–Meier plots using 2 genes (CYP3A4 and NOVA1) detected by the proposed method.

To investigate the performance of the proposed method in a comparison of the seven popular methods (*t*-test, Wilcoxon, SAM, LIMMA, WAD, RP, and FCROS), we directly applied this methods in this dataset to identify the DEGs. Figure 4a,b represents the Venn diagram of the top 50 DEGs detected by the *t*-test, SAM, LIMMA, and Proposed and WAD, RP, FCROS, and Proposed, respectively. We used the Benjamin–Hochberg (BH) method to adjust the *p*-values for all methods. From Figure 4a, we notice that there are 34 DEGs that are common to all the four methods (*t*-test, SAM, LIMMA, and Proposed). The proposed method identified 15% outlying genes using the *β*-weight function. Figure 4c shows the scatter plot of ordered smallest *β*-weight for each gene with its index and the Figure 4d show the histogram of *β*-weights. In both figures the outlier genes are indicated by red color. From Figure 4a,b we observe that the proposed method shares more genes with the other methods. The proposed method identified two (2) DE genes (CYP3A4 and NOVA1) that were not detected by the other three methods (*t*-test, SAM, and LIMMA). Among these two genes, CYP3A4 is outlying gene. To explore the biological functions of these genes (CYP3A4 and NOVA1), we used WebGestalt2 software package [32]. From this website we obtained GO (Gene Ontology), KEGG pathway, and disease association results. From the GO database we revealed that these genes are involved in different biological processes, such as metabolic process, biological regulation, cell communication, response to stimulus, etc., and different molecular processes such as nucleic site binding, protein binding, lipid binding, oxygen binding, ion binding, and so on (see Files S1 and S2 (.xls) in supplementary file 2). Using the KEGG database, we found that these two genes are significantly enriched in linoleic acid metabolism, drug metabolism, steroid hormone biosynthesis, retinol metabolism, bile secretion, and metabolic pathways (see Table 4). The disease association results of these two genes are summarized in Table 5 and revealed that these genes are associated with some cancer-related diseases such as prostate cancer, osteosarcoma, mammary neoplasms etc. The last column of the tables represents the adjusted *p*-values. The *p*-values were obtained from the hypergeometric test and adjusted by Benjamin–Hochberg method. Therefore, we may conclude that these two genes (CYP3A4 and NOVA1) identified by the proposed method may be cancer biomarker as they are involved in different cancer-related pathways. A protein–protein interaction (PPI) is constructed in Figure 5 to show the relationship interactions between the DEGs using BioGRID web server database [33]. In addition, we performed a prognostic power analysis through multivariate Cox regresssion as implemented in SurvExpress [34] using an independent RNA-seq dataset obtained from The Cancer Genome Atlas (TGCA). This dataset consist of 283 samples. The estimated survival probabilities are represented by Kaplan–Meier plot in Figure 6. From this analysis we demonstrate that the combined effect of these 2 genes has prognostic capability in HNC with a hazards ratio of 1.59 and log-rank *p*-value = 0.012. Furthermore, to propose candidate drugs and association of drugs with the identified genes, we used GLAD4U and Drug bank database. 12 statistically significant drugs were obtained interacting two genes (adjusted *p*-value < 0.05) (Table 6). The identified drugs were (darunavir, Cremophor EL, nilvadipine, alprazolam, felodipine, triazolam, androgen, progestogen and estrogen in combination, quinine and derivatives, desloratadine, acetaminophen glucuronide, clarithromycin, erythromycin, and paliperidone).

## 4. Discussion

Identification of DEGs across two or more biological conditions is an important task for microarray data analysis. The simplest method for detecting DEGs with biological significance is fold change (FC). However this approach does not take variability into account. Therefore, the statistical *t*-test is extensively used for identification of DEGs by considering the variability in the gene expression in microarray data. However, *t*-test suffers from multiple testing problems and small sample sizes. The significance analysis of microarrays (SAM) is very popular method for detecting the DEGs. SAM explicitly addressed the issues of *t*-test. However, it is not robust against outliers. Nowadays, the combination of FC and *p*-value-based methods is proven better than FC or *p*-value-based methods for gene selection and ranking. There are some methods that combine the *p*-value and FC value such as TREAT, *π*-value, RP, and FCROS. In TREAT a threshold was introduced between the gaps of average in the Student’s *t*-test [20]. A score was calculated by combining FC with a two samples statistical test *p*-value, which they called *π*-value [23]. However, the problems of multiple tests arise using these tests since these methods calculate their probabilities independently for each gene [21]. In RP the FCs are ranked in deceasing order and a product of the ranks (RP) for each gene is calculated. FCROS method has solved the problems of multiple tests [22]. They exploited variations in calculated FC levels using combinatorial pairs of control/test samples. However, the performance of these methods also deteriorated with the lower number of replicates in the biological conditions, in the presence of outliers. Therefore, in this paper, we have proposed a new hybrid robust SAM-FC approach by combining rank of FC values and rank of *p*-values based on SAM statistic using minimum *β*-divergence estimators for paired data to solve the aforementioned problems. The proposed method unifies both classical and robust estimate in a way such that in absence of outliers the MLEs are used and in presence of outliers minimum *β*-divergence estimators are used to obtain FC-values and *p*-values from SAM statistic under the null hypothesis of zero mean. The simulated and real Spike gene expression profiles analysis results showed that all eight methods (*t*-test, Wilcoxon, SAM, LIMMA, WAD, RP, FCROS, and Proposed) performed almost similar in the absence of outliers for both small and large sample cases. Four methods (Wilcoxon, RP, FCROS, and Proposed) perform better compared to the other four methods (*t*-test, SAM, LIMMA, and WAD) for large sample cases in the presence of outliers. However, the proposed method performs much better than the other seven methods (*t*-test, Wilcoxon, SAM, LIMMA, WAD, RP, and FCROS) for small sample case in presence of outliers. From HNSCC gene expression profiles, the proposed method detected additional two (2) DEGs that were not detected by three traditional methods (*t*-test, SAM, and LIMMA). Using the KEGG pathway enrichment analysis, we revealed that these two additional DEGs (NOVA1 and CYP3A4) are involved in some important pathways such as linoleic acid metabolism, drug metabolism, steroid hormone biosynthesis, and metabolic pathways. Using survival analysis through the Kaplan–Meier curve we have found that combined effect of these two genes has prognostic capability and they might be promising biomarker of HNC. In addition, the present study also revealed association of these two genes with 12 candidate drugs. Therefore, we think that these 12 candidate drugs might be potential therapeutic targets and precision therapeutic strategy in cancer. We have designed the proposed method especially for paired samples or equal sample sizes of two conditions. For unequal samples we recommend to use our previous paper, robust SAM [26]. Our future interest is to apply the proposed method in other cancer datasets.

## 5. Conclusions

In this paper we proposed a new hybrid robust SAM-FC approach to obtain a robust score using rank of FC values and rank of *p*-values based on SAM statistic by minimum *β*-divergence estimators. We used *β* = 0.2 for the measure of outlier detection. The proposed method uses the *β*-weight function to unify both classical and robust estimates to calculate the robust score. We examined the performance of proposed method in a comparison of some popular methods (*t*-test, Wilcoxon, SAM, LIMMA, WAD, RP, and FCROS) using both simulated and real gene expression profiles for both small-and large sample cases. We observed that the proposed method outperforms other methods for small sample case in presence of outliers and it keeps almost equal performance with other robust methods (Wilcoxon, RP, and FCROS) otherwise. Both types of data analysis results showed that the performance of the proposed method improves over the other methods for both small-and large sample cases. Therefore, our proposal is to use the proposed method instead of existing methods to obtain the better performance for identification of cancer biomarkers and candidate drugs.

## Figures and Tables

**Table 1:** Performance evaluation based on simulated gene expression profiles using data type 1.

**Average Performance Results for Small Sample Case (*n*_1_*= n*_2_*=* 3)**								
**Methods**	**TPR**	**FPR**	**TNR**	**FNR**	**MER**	**FDR**	**AUC**	**pAUC**
**t-test**	0.461 (0.132)	0.017 (0.027)	0.983 (0.973)	0.539 (0.868)	0.033 (0.052)	0.538 (0.864)	0.459 (0.131)	0.090 (0.026)
**Wilcoxon**	0.889 (0.141)	0.003 (0.026)	0.997 (0.974)	0.111 (0.859)	0.007 (0.052)	0.112 (0.854)	0.889 (0.141)	0.178 (0.028)
**SAM**	0.802 (0.145)	0.006 (0.026)	0.994 (0.974)	0.198 (0.855)	0.012 (0.051)	0.202 (0.850)	0.802 (0.145)	0.160 (0.029)
**Limma**	0.924 (0.145)	0.002 (0.026)	0.998 (0.974)	0.076 (0.855)	0.005 (0.051)	0.081 (0.850)	0.924 (0.145)	0.185 (0.029)
**WAD**	0.785 (0.002)	0.007 (0.031)	0.993 (0.969)	0.215 (0.998)	0.013 (0.060)	0.214 (0.998)	0.785 (0.002)	0.157 (0.000)
**RP**	0.926 (0.010)	0.002 (0.031)	0.998 (0.969)	0.074 (0.990)	0.005 (0.060)	0.080 (0.989)	0.926 (0.010)	0.185 (0.002)
**FCROS**	0.909 (0.146)	0.003 (0.026)	0.997 (0.974)	0.091 (0.854)	0.006 (0.051)	0.096 (0.849)	0.909 (0.146)	0.182 (0.029)
**Proposed**	0.924 (0.855)	0.002 (0.010)	0.998 (0.990)	0.076 (0.145)	0.005 (0.019)	0.081 (0.153)	0.924 (0.850)	0.185 (0.166)
***p*-value**	**0.000 (0.000)**	**0.000 (0.040)**	**0.000 (0.000)**	**0.000 (0.000)**	**0.000 (0.000)**	**(0.000) (0.000)**	**0.000 (0.000)**	**0.000 (0.000)**
**Average Performance Results for Small Sample Case (*n*_1_*= n*_2_*=* 15)**								
**Methods**	**TPR**	**FPR**	**TNR**	**FNR**	**MER**	**FDR**	**AUC**	**pAUC**
**t-test**	0.927 (0.144)	0.003 (0.026)	0.997 (0.974)	0.073 (0.856)	0.005 (0.051)	0.089 (0.852)	0.927 (0.144)	0.185 (0.028)
**Wilcoxon**	0.927 (0.893)	0.003 (0.004)	0.997 (0.996)	0.073 (0.107)	0.005 (0.007)	0.089 (0.122)	0.927 (0.893)	0.185 (0.178)
**SAM**	0.927 (0.144)	0.003 (0.026)	0.997 (0.974)	0.073 (0.856)	0.005 (0.051)	0.089 (0.521)	0.927 (0.144)	0.185 (0.029)
**Limma**	0.927 (0.145)	0.003 (0.026)	0.997 (0.974)	0.073 (0.855)	0.005 (0.051)	0.089 (0.851)	0.927 (0.145)	0.185 (0.029)
**WAD**	0.909 (0.012)	0.006 (0.031)	0.993 (0.969)	0.090 (0.988)	0.011 (0.059)	0.102 (0.990)	0.909 (0.011)	0.181 (0.002)
**RP**	0.927 (0.926)	0.003 (0.003)	0.997 (0.997)	0.073 (0.074)	0.005 (0.005)	0.089 (0.090)	0.927 (0.926)	0.185 (0.185)
**FCROS**	0.927 (0.927)	0.003 (0.003)	0.997 (0.997)	0.073 (0.073)	0.005 (0.005)	0.089 (0.089)	0.927 (0.927)	0.185 (0.185)
**Proposed**	0.927 (0.927)	0.003 (0.003)	0.997 (0.997)	0.073 (0.073)	0.005 (0.005)	0.089 (0.089)	0.927 (0.927)	0.185 (0.185)
***p*-value**	**0.352 (0.000)**	**0.989 (0.000)**	**0.999 (0.000)**	**0.820 (0.000)**	**0.983 (0.000)**	**0.970 (0.000)**	**0.352 (0.000)**	**0.999 (0.000)**

Average performance results of eight methods (*t*-test, SAM, LIMMA, Wilcoxon, WAD, RP, FCROS, and Proposed) based on 100 datasets of data type 1 for both small and large sample cases *n*_1_ = *n*_2_ = 3 and 15. Each dataset for each case included 300 true DEGs. The performance measures TPR, FPR, TNR, FNR, FDR, MER, AUC, and pAUC were calculated for each methods based on top 300 estimated DEGs considering the rest of 9700 genes were EEGs. The performance measure pAUC was calculated at FPR = 0.2 for each method for each dataset. The values inside the parenthesis (.) indicate the average performance results in presence of one or two outlying sample across the genome.

**Table 2:** Performance evaluation based on spike gene expression profiles using data type 2 for sample size *n*_1_ = *n*_2_ = 9.

Performance Results for Sample Size *n*_1_ = *n*_2_ = 9								
Methods	TPR	TNR	FPR	FNR	FDR	MER	AUC	pAUC
**t-test**	0.818 (0.217)	0.979 (0.909)	0.021 (0.091)	0.182 (0.783)	0.182 (0.783)	0.038 (0.163)	0.816 (0.216)	0.161 (0.043)
**Wilcoxon**	0.810 (0.540)	0.978 (0.947)	0.022 (0.053)	0.190 (0.460)	0.190 (0.460)	0.040 (0.096)	0.805 (0.534)	0.158 (0.102)
**SAM**	0.833 (0.215)	0.981 (0.909)	0.019 (0.091)	0.167 (0.785)	0.167 (0.785)	0.035 (0.163)	0.832 (0.214)	0.165 (0.042)
**LIMMA**	0.830 (0.217)	0.980 (0.909)	0.020 (0.091)	0.170 (0.783)	0.170 (0.783)	0.035 (0.163)	0.828 (0.216)	0.164 (0.043)
**WAD**	0.831 (0.297)	0.980 (0.918)	0.020 (0.082)	0.169 (0.703)	0.169 (0.703)	0.035 (0.146)	0.830 (0.284)	0.165 (0.046)
**RP**	0.824 (0.743)	0.980 (0.970)	0.020 (0.030)	0.176 (0.257)	0.176 (0.257)	0.037 (0.053)	0.822 (0.741)	0.163 (0.146)
**FCROS**	0.834 (0.799)	0.981 (0.977)	0.019 (0.023)	0.166 (0.201)	0.166 (0.201)	0.035 (0.042)	0.833 (0.798)	0.166 (0.158)
**Proposed**	0.837 (0.832)	0.981 (0.981)	0.019 (0.019)	0.163 (0.168)	0.163 (0.168)	0.032 (0.035)	0.837 (0.831)	0.170 (0.165)
***p*-value**	**0.807 (0.000)**	**0.999 (0.000)**	**0.999) (0.000)**	**0.766 (0.000)**	**0.766 (0.000)**	**0.991 (0.000)**	**0.610 (0.000)**	**0.998 (0.000)**

The summary statistics (TPR, TNR, FPR, FNR, FDR, MER, AUC, and pAUC) were calculated based on top 1944 DEGs estimated by different methods (*t*-test, Wilcoxon, SAM, LIMMA, WAD, RP, FCROS, and Proposed). The results inside the parenthesis (.) indicate the summary statistics in presence of one outlying sample across the genome.

**Table 3:** Performance evaluation based on spike gene expression profiles using data type 2 for small sample cases *n*_1_ = *n*_2_ = 3.

Average Performance Results for Small Sample Case *n*_1_ = *n*_2_ = 3								
Methods	TPR	TNR	FPR	FNR	FDR	MER	AUC	pAUC
**t-test**	0.6939 (0.2253)	0.9645 (0.9102)	0.0355 (0.0898)	0.3061 (0.7747)	0.3061 (0.7747)	0.0636 (0.1610)	0.6888 (0.2234)	0.1337 (0.0432)
**Wilcoxon**	0.3405 (0.2238)	0.9235 (0.9100)	0.0765 (0.0900)	0.6595 (0.7762)	0.6595 (0.7762)	0.1371 (0.1613)	0.3278 (0.2178)	0.0553 (0.0388)
**SAM**	0.7701 (0.1456)	0.9733 (0.9009)	0.0267 (0.0991)	0.2299 (0.8544)	0.2299 (0.8544)	0.0478 (0.1776)	0.7683 (0.1445)	0.1522 (0.0281)
**LIMMA**	0.7675 (0.2068)	0.9730 (0.9080)	0.0270 (0.0920)	0.2325 (0.7932)	0.2325 (0.7932)	0.0483 (0.1649)	0.7659 (0.2060)	0.1519 (0.0405)
**WAD**	0.7639 (0.2850)	0.9726 (0.9171)	0.0274 (0.0829)	0.2361 (0.7150)	0.2361 (0.7150)	0.0491 (0.1486)	0.7627 (0.2715)	0.1516 (0.0435)
**RP**	0.7711 (0.1898)	0.9735 (0.9060)	0.0265 (0.0940)	0.2289 (0.8102)	0.2289 (0.8102)	0.0476 (0.1684)	0.7696 (0.1796)	0.1527 (0.0277)
**FCROS**	0.7685 (0.3853)	0.9732 (0.9287)	0.0268 (0.0713)	0.2315 (0.6147)	0.2315 (0.6147)	0.0481 (0.1278)	0.7671 (0.3757)	0.1523 (0.0675)
**Proposed**	0.7716 (0.7582)	0.9735 (0.9720)	0.0265 (0.0280)	0.2284 (0.2418)	0.2284 (0.2418)	0.0475 (0.0502)	0.7702 (0.7565)	0.1529 (0.1499)
***p*-value**	0.000 (0.000)	0.000 (0.000)	0.000 (0.000)	0.000 (0.000)	0.000 (0.000)	0.000 (0.000)	0.000 (0.000)	0.000 (0.000)

Average performance results by eight methods (*t*-test, Wilcoxon, SAM, LIMMA, WAD, RP, FCROS, and Proposed) based on 100 bootstrap datasets of data type 2 for small sample case (*n*_1_ ***=*** *n*_2_ = 3). In each bootstrap dataset, 3 expression data points are selected from each condition for each gene from original dataset. The summary statistics (TPR, TNR, FPR, FNR, FDR, MER, AUC, and pAUC) were calculated based on top 1944 DEGs estimated by each method. The results inside the parenthesis (.) indicate the summary statistics in presence of one outlying sample across the genome.

**Table 4:** KEGG pathways for the two (2) differentially expressed (DE) genes identified by the proposed method only.

KEGG ID	Pathway Name	Adjusted *p*-Value
hsa00591	Linoleic acid metabolism	0.004
hsa00983	Drug metabolism-other enzymes	0.006
hsa00140	Steroid hormone biosynthesis	0.008
hsa00830	Retinol metabolism	0.009
hsa00982	Drug metabolism-cytochrome P450	0.009
hsa04976	Bile secretion	0.009
hsa00980	Metabolism of xenobiotics by cytochrome	0.010
hsa05204	Chemical carcinogenesis	0.010
hsa01100	Metabolic pathways	0.010

**Table 5:** Disease association results of two (2) genes identified by proposed method only.

ID	Disease Name	Adjusted *p*-Value
umls:C0040479	Torsades de Pointes	0.0005
umls:C0019196	Hepatitis C	0.0016
umls:C0029463	Osteosarcoma	0.0041
umls:C1458155	Mammary Neoplasms	0.040
umls:C0033578	Prostatic Neoplasms	0.051

**Table 6:** Candidate drugs for the two (2) DE genes identified by the proposed method obtained from GLAD4U and drug bank databases.

ID	Name of the Drug	Adjusted *p*-Value
PA163522472	darunavir	7.11 × 10^−4^
PA165111677	Cremophor EL	7.11 × 10^−4^
PA165958385	nilvadipine	7.11 × 10^−4^
PA448333	alprazolam	7.11 × 10^−4^
PA449591	felodipine	7.11 × 10^−4^
PA451753	triazolam	7.11 × 10^−4^
PA164712364	Androgen, progestogen and estrogen in combination	8.53 × 10^−4^
PA164713223	Quinine and derivatives	8.53 × 10^−4^
PA164776964	desloratadine	8.53 × 10^−4^
PA165983955	acetaminophen glucuronide	8.53 × 10^−4^
DB01211	Clarithromycin	2.22 × 10^−3^
DB00199	Erythromycin	3.11 × 10^−3^
DB01267	Paliperidone	7.55 × 10^−3^

## Supplementary Materials

The following are available online at https://www.mdpi.com/1010-660X/55/6/269/s1. **Supplementary file 1:** Figure S1: Volcano plot in presence of 10% outliers for simulated dataset. Figure S2: Predicted distribution of *β*-weights using data type 1 for small sample case *n*1 = *n*2 = 3 with m = 2 conditions. Figure S3: Performance evaluation using boxplot of AUC values estimated by different methods for data type 1. Figure S4: Performance evaluation using boxplot of MER values estimated by different methods for data type 1. Figure S5: Plot of FNR versus FPR estimated by different methods using data type 1 with m = 2 conditions. Figure S6: ROC curve produced by different methods using data type 1 with m = 2 conditions. Figure S7: Results of spike data analysis. Supplementary file 2: Head and Neck Cancer and Spike-in dataset results. Supplementary file 3: R code of the proposed procedure. Software: The R-package for implementing the proposed methodology can be downloaded at http://www.bbcba.org/softwares/rSAM-FC.zip.
