# Supplementary material for: A Robust Approach for Identification of Cancer Biomarkers and Candidate Drugs
Source: Medicina (Kaunas). 2019 Jun 11;55(6):269. doi: 10.3390/medicina55060269 (PMC6631768; doi:10.3390/medicina55060269)
Supplement: Supplementary file 1 [file medicina-55-00269-s001.zip › Supplementary_file/Supplementary file 2/Table A1.docx]

**Table A1: Comparative results for identification of number of true DE genes for data type 2 by eight methods.**

| Declared True  DE genes | **Methods** | | | | | | | |
| --- | --- | --- | --- | --- | --- | --- | --- | --- |
|  | **For sample size n_1_=n_2_=9** | | | | | | | |
| 1944 | **t-test** | **Wilcoxon** | **SAM** | **LIMMA** | **WAD** | **RP** | **FCROS** | **Proposed** |
|  | 1591  (422) | 1574  (1049) | 1620  (417) | 1613  (422) | 1616  (577) | 1601  (1445) | 1621  (1553) | 1628  (1618) |
|  | **For sample size n_1_=n_2_=3** | | | | | | | |
|  | **t-test** | **Wilcoxon** | **SAM** | **LIMMA** | **WAD** | **RP** | **FCROS** | **Proposed** |
|  | 1349  (438) | 662  (435) | 1497  (283) | 1492  (402) | 1485  (554) | 1499  (369) | 1494  (749) | 1500  (1474) |

Performance evaluation for detection of number of true DE genes by eight methods (t-test, Wilcoxon, SAM, LIMMA, WAD, RP, FCROS and Proposed) based on spike gene expression profiles using data type 2. The results inside the parenthesis (.) indicate the number of genes estimated by eight methods that were common with declared DE genes, in presence of one outlying sample across the genome
