# Supplementary figures and images for: A Robust Approach for Identification of Cancer Biomarkers and Candidate Drugs
Source: Medicina (Kaunas). 2019 Jun 11;55(6):269. doi: 10.3390/medicina55060269 (PMC6631768; doi:10.3390/medicina55060269)

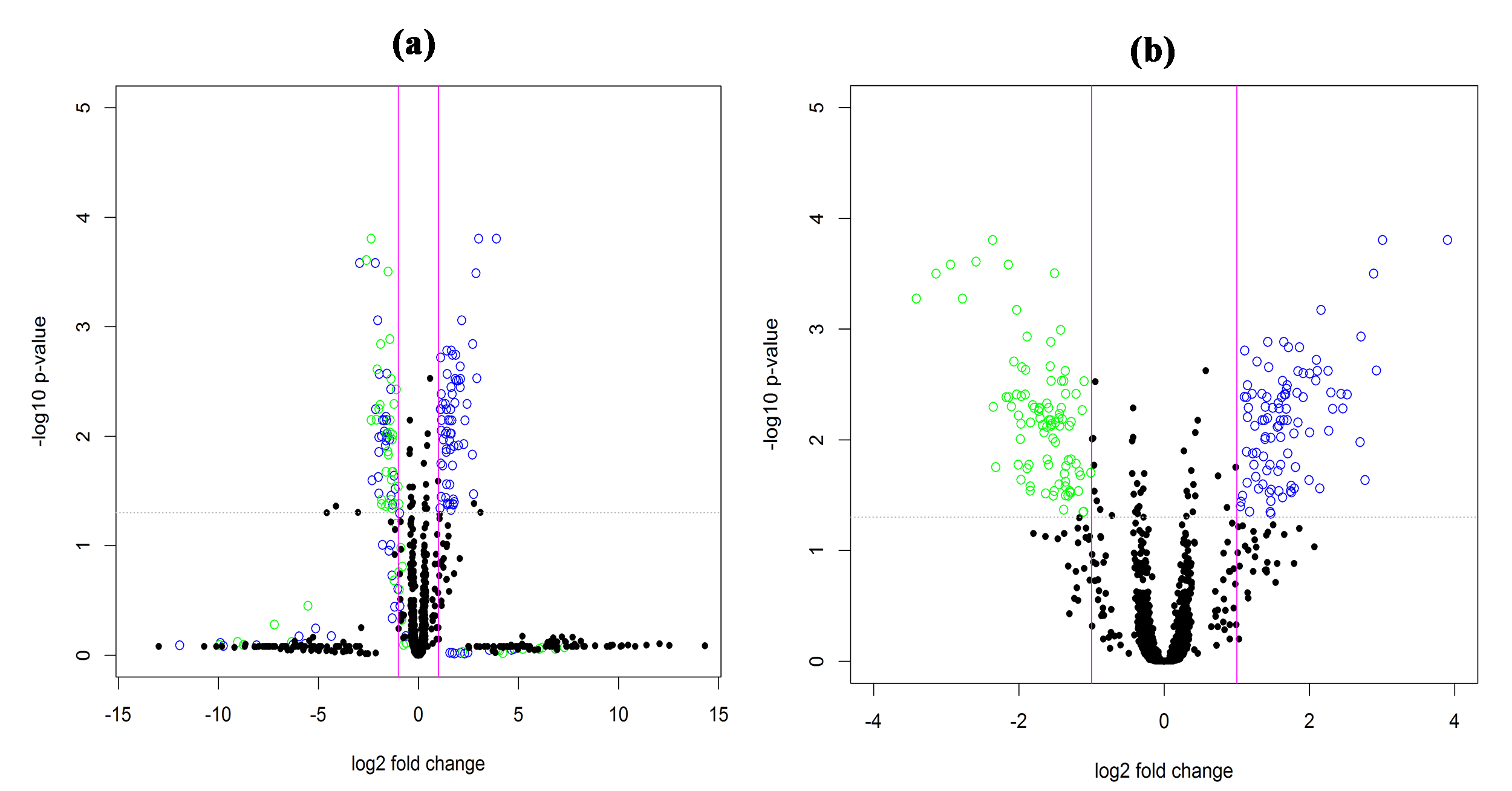

Supplement: Supplementary file 1 [file medicina-55-00269-s001.zip › Supplementary_file/Supplementary file 1/S1.tif]

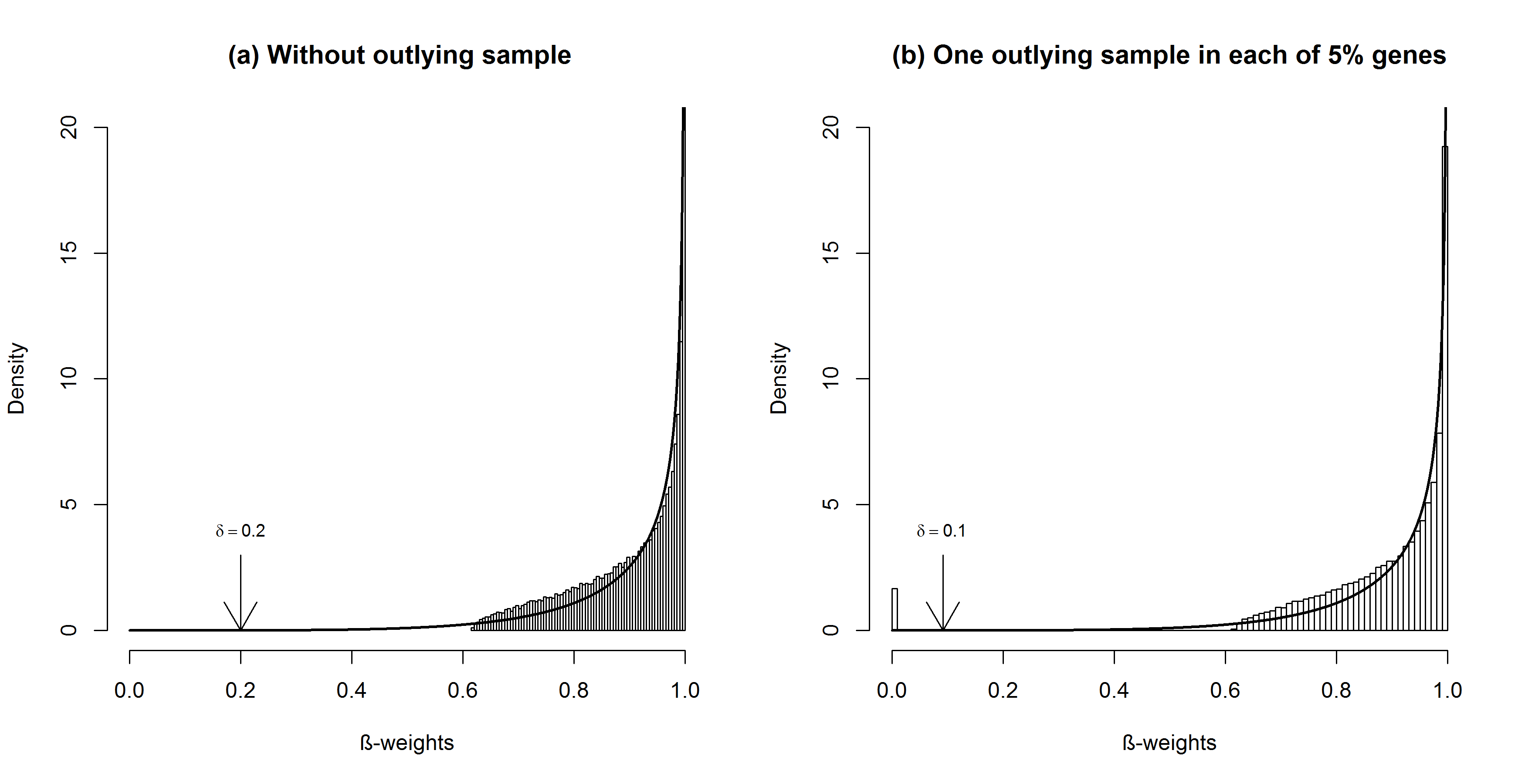

Supplement: Supplementary file 1 [file medicina-55-00269-s001.zip › Supplementary_file/Supplementary file 1/S2.tiff]

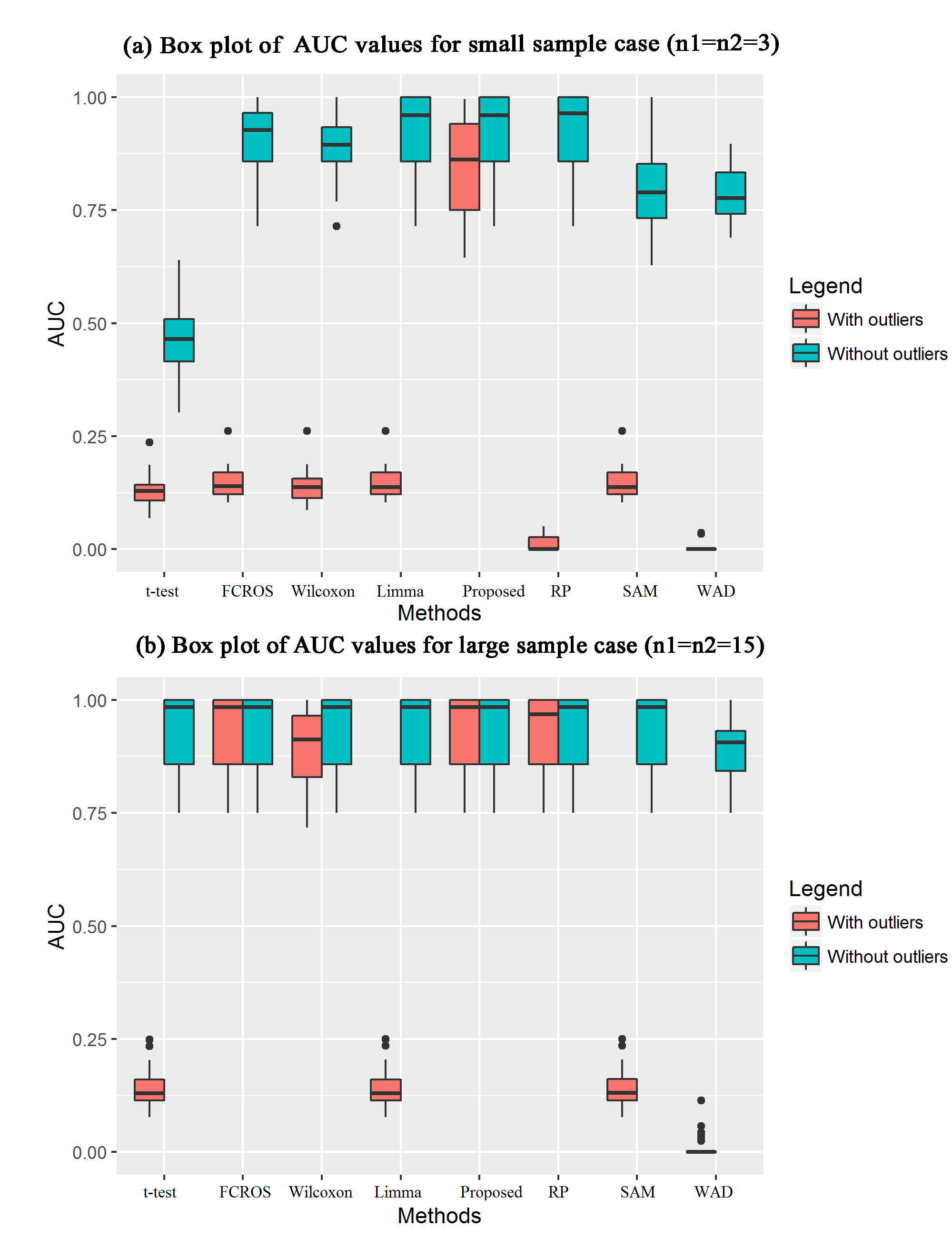

Supplement: Supplementary file 1 [file medicina-55-00269-s001.zip › Supplementary_file/Supplementary file 1/S3.tif]

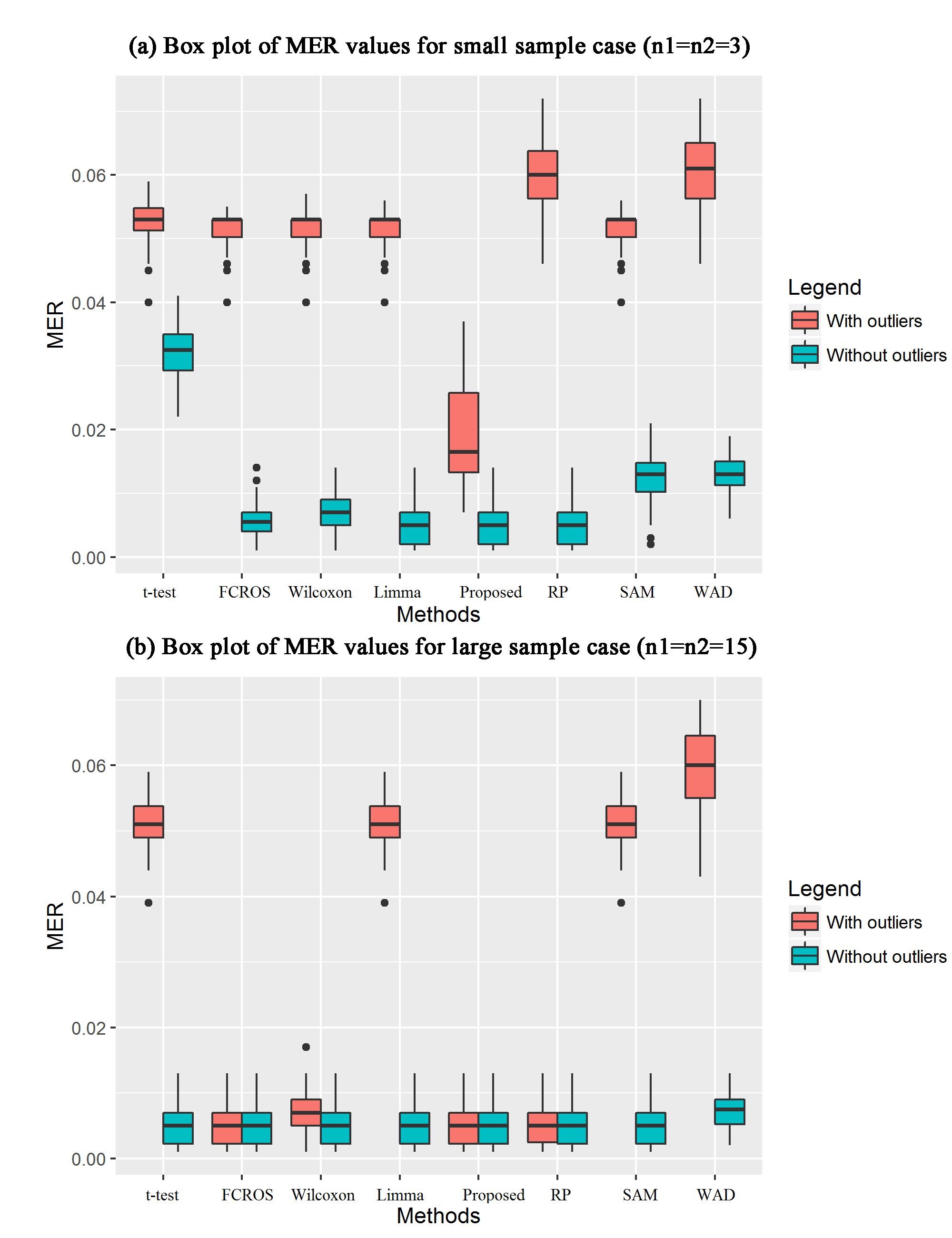

Supplement: Supplementary file 1 [file medicina-55-00269-s001.zip › Supplementary_file/Supplementary file 1/S4.tif]

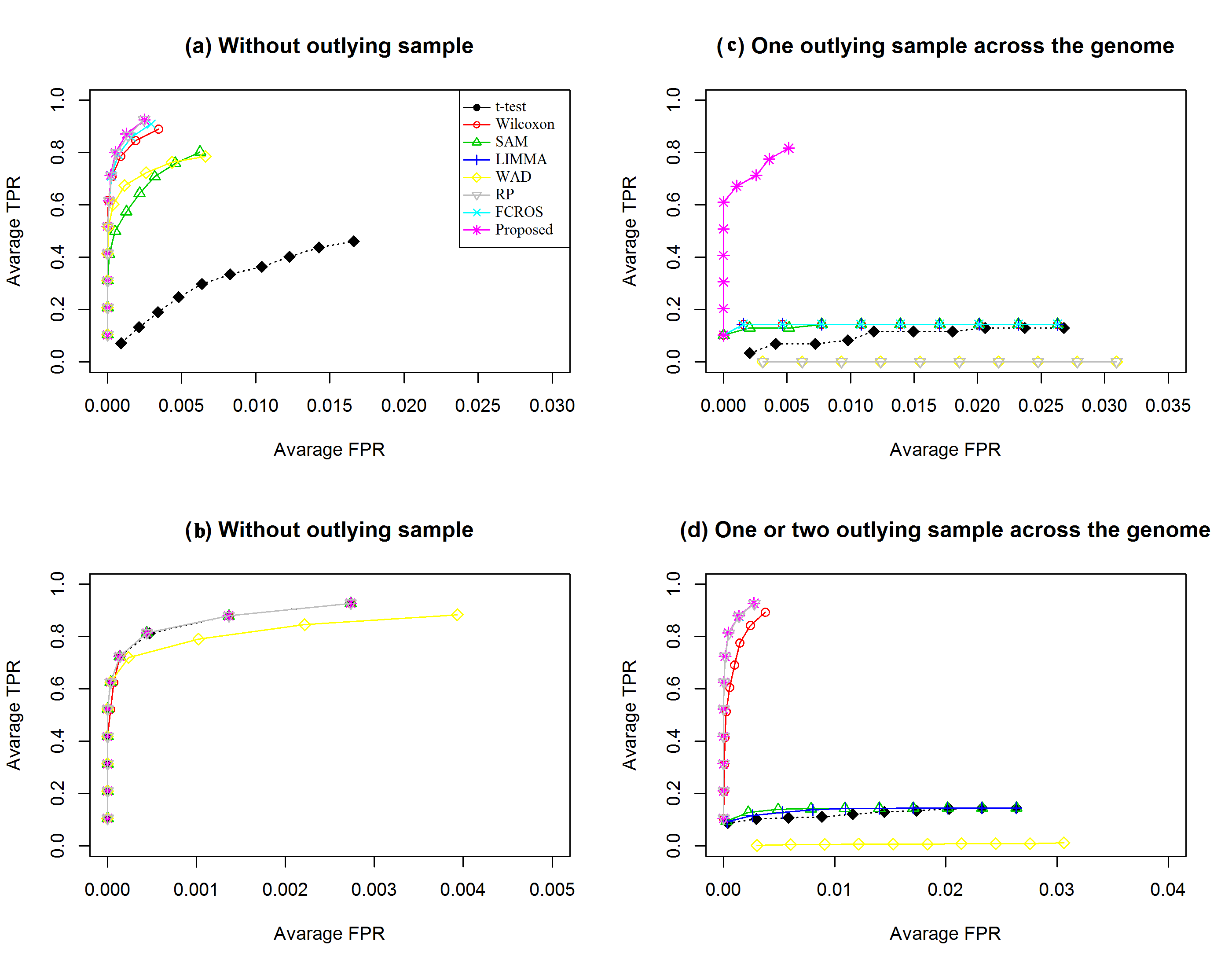

Supplement: Supplementary file 1 [file medicina-55-00269-s001.zip › Supplementary_file/Supplementary file 1/S5.tiff]

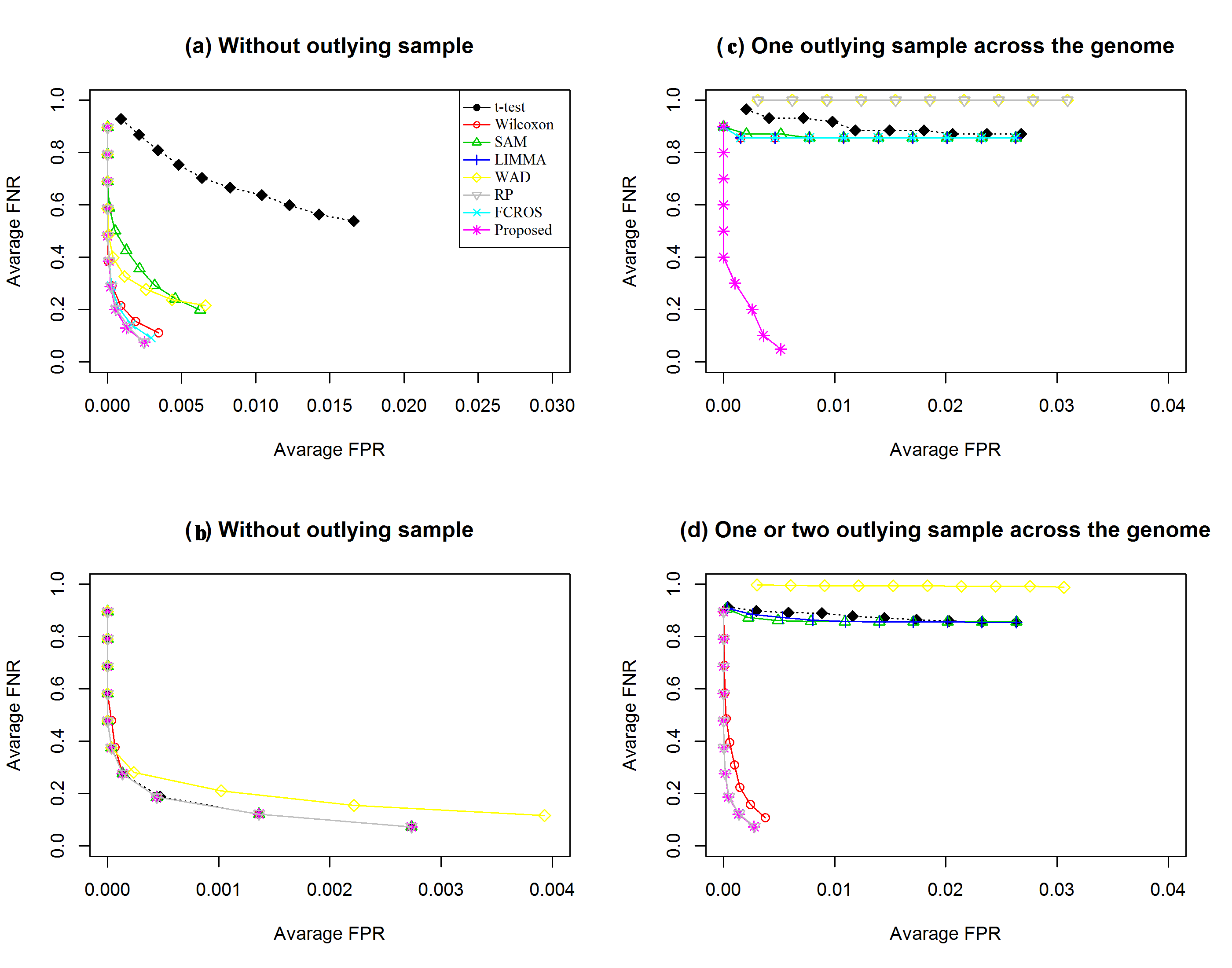

Supplement: Supplementary file 1 [file medicina-55-00269-s001.zip › Supplementary_file/Supplementary file 1/S6.tiff]

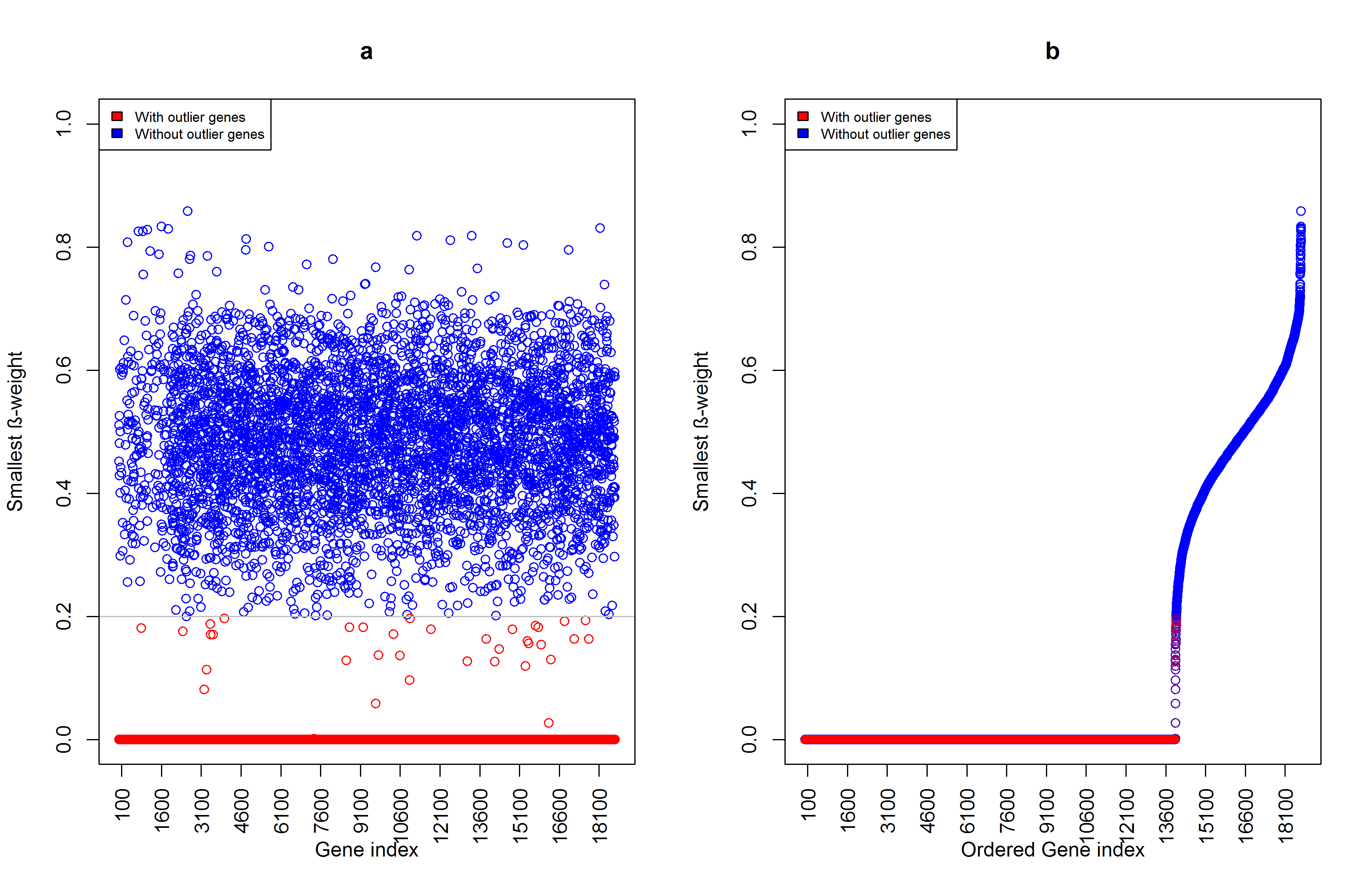

Supplement: Supplementary file 1 [file medicina-55-00269-s001.zip › Supplementary_file/Supplementary file 1/S7.tiff]
